# Immunomodulatory effect of probiotic exopolysaccharides in a porcine in vitro co-culture model mimicking the intestinal environment on ETEC infection

**DOI:** 10.1007/s11259-023-10237-4

**Published:** 2023-10-24

**Authors:** Zuzana Kiššová, Dagmar Mudroňová, Róbert Link, Ľudmila Tkáčiková

**Affiliations:** 1 Department of Morphological Disciplines, University of Veterinary Medicine and Pharmacy in Košice, Komenského 73, 041 81 Košice, Slovakia; 2 Department of Microbiology and Immunology, University of Veterinary Medicine and Pharmacy in Košice, Komenského 73, 041 81 Košice, Slovakia; 3 Clinik of Swine, University Veterinary Hospital, University of Veterinary Medicine and Pharmacy in Košice, Komenského 73, 041 81 Košice, Slovakia

**Keywords:** Co-culture, IPEC-J2, moDCs, EPS, ETEC, Cytokines, qPCR

## Abstract

The aim of this study was to evaluate the immunomodulatory effect of EPS-L26 isolated from the probiotic strain *Lactobacillus* (*Limosilactobacillus*) *reuteri* L26 Biocenol™, in a model of infection with an enterotoxigenic *E. coli* (ETEC) by establishing monocultures consisting of the IPEC-J2 cell line or monocyte-derived dendritic cells (moDCs) and creating a 3D model of cell co-cultures established with IPEC-J2 cells and moDCs. The immunomodulatory and immunoprotective potential of used EPS-L26 was confirmed in monocultures in an experimental group of pretreated cells, where our study showed that pretreatment of cells with EPS-L26 and subsequent exposure to infection resulted in significantly down-regulated mRNA levels of genes encoding inflammatory cytokines compared to ETEC challenge in single cell cultures (in IPEC-J2, decreased mRNA levels for TNF-α, IL-6, IL-1β, IL-12p35; in moDCs, decreased mRNA levels for IL-1β). Similar to monocultures, we also demonstrated the immunostimulatory potential of the ETEC strain in the co-culture model on directly treated IPEC-J2 cells cultivated on insert chambers (apical compartment) and also on indirectly treated moDCs cultivated in the lower chamber (basolateral compartment), however in the co-culture model the expression of inflammatory cytokines was attenuated at the mRNA level compared to monocultures. Pretreatment of the cells on the insert chambers pointed to the immunoprotective properties of EPS-L26, manifested by decreased mRNA levels in both cell lines compared to ETEC challenge (in IPEC-J2 decreased mRNA levels for IL-12p35; in moDCs decreased mRNA levels for IL-1β, IL-6). Our results suggest intercellular communication via humoral signals derived from IPEC-J2 cells by influencing the gene expression of indirectly treated moDC cells located in the basolateral compartment.

## Introduction

Lactic acid bacteria (LAB) are well-known beneficial bacteria that can provide various health benefits to humans and animals. Certain strains of probiotic bacteria produce various biologically active substances, with particular attention being paid to exopolysaccharides (EPS) due to their significant and diverse beneficial effects on the host (Pérez-Ramos et al. 2016). By adhering to the intestinal epithelial surface, probiotic bacteria can modulate several intestinal functions including digestion, metabolism, and innate mucosal immunity mechanisms. Thanks to these properties, they can be used to prevent and alleviate the course of various gastrointestinal diseases, thus appearing to be suitable as a substitute for antimicrobial preparations and thus ensuring the maintenance of health, prevention and treatment of gastrointestinal infections in humans and animals.

The intestinal epithelium plays a key role in maintaining immune homeostasis in the gut, not only as an indispensable barrier between the luminal contents of the gut and the underlying immune cells, but also as an active player in both—maintaing tolerance to the microflora and food antigens and in fighting pathogens. Dendritic cells (DC) are considered the gatekeepers of the immune system and contact between DC and the gut microflora is essential for proper immune development and regulation. In the healthy state, subepithelial DCs make direct contact with the microflora by sampling luminal bacterial antigens (Allaire et al. 2018) or, in addition, directly interact with bacteria that have gained access via specialised epithelial cells called M cells (Zeuthen et al. 2008).Probiotic strains which produce EPS form a biofilm layer on the intestinal mucosa, protecting it from damage caused by pathogens or toxins. The ability of EPS molecules to reduce or prevent the binding of infectious bacteria to the intestinal surface contributes significantly to reducing their adhesion and thus eliminating their pathogenic effects (Castro-Bravo et al. 2018).

Enterotoxigenic *Escherichia coli* (ETEC) strains are gram-negative enteric pathogens that are the most common cause of diarrhoea in humans and animals (Xu et al. 2023). ETEC is also a major cause of diarrhoea in neonatal piglets and weaned piglets, resulting in significant economic losses to farmers (losses due to mortality, morbidity, reduced growth rate, and medication costs). Long-term use of antibiotics to control bacterial pathogens promotes the development of antibiotic resistance and the spread of resistance genes. The use of antibiotics in livestock not only increases the risk of pathogenic bacteria developing resistance in the animal ‘s fecal flora, but also poses a high risk of resistance transmission from animals to humans through contaminated food (Kumar et al. 2020).

Since bacterial exopolysaccharides contribute to the immunomodulation of innate immune responses by interacting with dendritic cells and macrophages (Pérez-Ramos et al. 2016), the first aim of the present study was to determine whether exopolysaccharides purified from the probiotic strain *L. reuteri* L26 Biocenol™ could influence the inflammatory response of porcine intestinal epithelial cells IPEC-J2 and moDCs in vitro when "pretreated" prior to infection with ETEC bacteria, which represent serious pathogens in the swine industry, particularly in the post-weaning period (Fairbrother and Nadeau 2019). The second aim of the present study was to investigate whether the inflammatory response of moDC monocultures and IPEC-J2 cells changes depending on their mutual interference in an in vitro co-culture model.

## Material and methods

### Porcine intestinal epithelial cells

The IPEC-J2 cell line was kindly provided by J.J. Garrido, Department of Genetics and Animal Breeding at the University of Córdoba, Spain. Cells were cultivated as described previously according to the study by Kiššová et al. (2022). Medium was chaged 3 time per week. Passages between 25 -30 were included in the experiments.

For IPEC-J2 monoculture experiments, the following protocol was used: IPEC-J2 were seeded on 12-well plates (TPP, Switzerland) at a density of 1.5 × 10^4^/ cm^2^ and cultivated under a humidified atmosphere of 5% CO_2_ at 37 °C for 72 h. Cells were cultured in an IPEC-J2 medium supplemented with hydrocortisone (0.28 µM, Sigma-Aldrich) and ascorbic acid (5 µg/mL, Sigma-Aldrich) to prevent cell preactivation. 24 h before the experiments, the culture medium was changed to IPEC-J2 medium without any of the above supplementations (as well as without FBS and antibiotics). After overnight cultivation, the cells were washed twice with phosphate-buffered saline (PBS) and were used for the following experiments.

For IPEC-J2 co-culture experiments, the following protocol was used: IPEC-J2 cells were seeded on the top of collagenased cell culture inserts of a 12-well Transwell^®^ system (12 mm diameter, 1.12 cm^2^ growth surface area, 0.4 μm pore size; Costar, Corning BV, The Netherlands) at a density of 2.5 × 10^5^ cells per cell culture insert. Cells were cultivated under a humidified atmosphere of 5% CO_2_ at 37 °C for 15—22 days. The TEER passing through a monolayer of IPEC-J2 cells was measured using an EVOM3 device (WPI, USA) every third day after seeding. TEER values were adjusted against a blank (control well). Results are reported as Ω × cm^2^. The medium was changed every third day as follows, first aspirated from the bottom well (basolateral side), then from the insert (apical side), and replaced the media in the opposite order—apical (500 µL) then basolateral (1.5 mL).

### Monolayer permeability test using horseradish peroxidase

Horseradish peroxidase (HRP, Sigma-Aldrich, USA) was used to test the monolayer permeability of IPEC-J2 cells seeded on Transwell^®^-COL inserts according to the study by Geens and Niewold (2011) with small modifications. IPEC-J2 cells were seeded on Transwell^®^-COL inserts at a high density of 12 × 10^5^ cells/mL to effectively saturate the available area for attachment and were cultivated under a humidified atmosphere of 5% CO_2_ at 37 °C. Permeability testing was performed on days 5, 10, 15, and 20 of cell seeding. On the indicated days, the cell culture medium on the apical side was discarded and replaced with 500 µL of HRP solution (20 µg/mL), and the medium on the basolateral side was replaced with 1.5 mL of Hanks' balanced salt solution (Sigma-Aldrich, USA). Plates were further incubated at 37 °C and samples (50 µL) were taken from the bottom well every 15 min. The samples were then incubated for 20 min at room temperature with 50 µL of ABTS substrate (KPL, USA), and the OD was measured at 450 nm using a Synergy HTX Multi-Mode Reader (Agilent, USA).

### Generation of monocyte-derived dendritic cells (moDCs)

Blood was collected under sterile conditions from the supraorbital sinus of clinically healthy Landras x Large White pigs (10 to 12 weeks of age), kept at the Clinic of Swine, University of Veterinary Medicine and Pharmacy, Košice. The procedure of blood sampling was performed in accordance with the guidelines for animal welfare and was approved by the Ethics Committee for Animal Welfare, namely, “Ethics Committee for the approval of research involving animals by the legislative requirements applicable at the UVMP in Košice” (Ethics committee at the UVMP in Košice, permit No. EKVP/2023–04). Blood samples were collected in 50 mL tubes filled with 1.5% heparin prepared in PBS.

Mononuclear leukocytes (MNL) were purified by density gradient centrifugation (300 × g 20 min) from the heparinized blood diluted 1:1 in PBS, underlaid with 15 mL separation solution (LSM1077 "Lymphocyte Separation Medium"; PAA, Austria) in Leucosep™ tubes (Greiner-Bio-One, Austria). MNLs were collected from the interface, then transferred into new tubes, and washed three times with PBS. After the last centrifugation, the supernatant was discarded and the MNL pellet was resuspended in RPMI 1640 (Roswell Park Memorial Institute-1640; Sigma-Aldrich, USA) medium, the cell number and viability were determined using (0.4%) trypan blue.

Monocytes were subsequently isolated from the MNL suspension by using non-magnetic microparticles (S-pluriBead®; pluriSelect Life Science, Germany) labeled with an antibody-specific mouse IgG (BioLabs, UK) against porcine CD14 (clone MIL-2, kindly provided by RNDr. J. Šinkora, PhD., Department of Immunology and Gnotobiology, Academy of Sciences of the Czech Republic, Prague (Czech Republic)) according to the manufacturer’s instructions. The isolated monocytes were then resuspended in RPMI 1640 medium and their number and viability were determined using (0.4%) trypan blue.

For moDC monoculture experiments the following protocol was used: isolated monocytes were seeded in a 12-well plate (TPP, Switzerland) at a density of 4.5 × 10^5^ per cm^2^ and incubated in RPMI 1640 culture medium supplemented with glutamine (2 mmol/L; Lonza, Switzerland), FBS (10%; Lonza, Switzerland), gentamicin (1%; PAA, Austria) and recombinant porcine cytokines rpGM-CSF (20 ng/mL; R&D Systems, USA) and rpIL-4 (50 ng/mL; R&D Systems, USA) at temperature 37 °C in an atmosphere enriched with 5% CO_2_ for 5 days. This complete culture medium was named as moDC medium and was changed every other day. On day 4, the moDC medium was supplemented with hydrocortisone (0.28 µM, Sigma-Aldrich) and ascorbic acid (5 µg/mL, Sigma-Aldrich) to prevent cell pre-activation. 24 h prior to the experiment (day 5 of seeding), the culture medium was changed to moDC medium without FBS and antibiotic supplementation, but with the addition of the growth factors rpGM-CSF (20 ng/mL; R&D Systems, USA) and rpIL-4 (50 ng/mL; R&D Systems, USA). After overnight cultivation, the cells were washed twice with PBS and used for the following experiments.

### Immunostaining and flow cytometry

Cytometric analysis of moDC was performed regularly to analyse surface markers (CD14 molecules, MHC class II molecules, costimulatory molecules CD80 and CD86) during cell differentiation using BD FACS Canto™ flow cytometer with BD FACS Diva software (BD Biosciences, USA). The primary and secondary antibodies used for analysis are listed in Table 1. moDCs were incubated with primary antibodies for 20 min at room temperature, then washed with PBS and incubated with secondary antibodies (1:100 dilution; 50 µL per 10^5^ cells) for 15–20 min at room temperature. Surface marker expression was assessed immediately by flow cytometry. The level of expression was expressed as the geometric mean of fluorescence intensity (MFI).

**Table 1:** Antibodies used for moDC maturation and differentiation

Primary antibodies	Secondary antibodies	References
Anti-Porcine CD14	Anti-Mouse IgG2b FITC	RNDr. Jiří Šinkora, PhD
Anti-Porcine MHC Class II DQ	Anti-mouse IgG1 APC	AbD Serotec, USA
Human CD152(CTLA-4) Ig/Fusion Protein	Anti-mouse IgG2a PE	Ancell, USA

### Testing moDC activation using LPS

Bacterial LPS (100 ng/mL; LPS, *E. coli* O111:B4; Sigma-Aldrich, USA) was used as a positive control for moDC activation on day 5, and cells were incubated with LPS for an additional 48 h. The cells were then non-enzymatically dissociated from the culture wells using a cell dissociation solution (Sigma-Aldrich, USA) according to the manufacturer's instructions. Flow cytometric analysis was performed to evaluate the expression of surface markers (CD14 molecules, MHC class II molecules, costimulatory molecules CD80 and CD86).

### Characteristics of EPS-L26

The exopolysaccharide (EPS) isolated from *L. reuteri* L26 Biocenol™, prepared by Kšonžeková et al. (2016), was used for the experiments. This purified EPS-L26 is an α-D-glucan with a high molecular weight of 8.2 × 10^5^ Da. The structure of this EPS-L26 is formed by branched α-D-glucose homopolymers with (1 → 3) and (1 → 6) glycosidic linkages in a ratio of 1.3:1 (Kšonžeková et al. 2016).

### Enterotoxigenic* E. coli *11,501

The enterotoxigenic (ETEC) strain *E. coli* 11,501 (O149: K88 + , STb + , LT + ; β-hemolysis) was kindly provided by MVDr. M. Faldyna, PhD, Research Institute of Veterinary Medicine, Brno (Czech Republic). This strain was obtained from the intestinal tract of young piglets that died with symptoms of hemorrhagic gastroenteritis. ETEC were cultivated in Luria’s broth (LB; Sigma-Aldrich, USA) at 37 °C for 16–18 h with constant stirring (160 rpm). Overnight cultures of bacteria were used as inoculum in LB broth to prepare a 2 h fresh culture.

The bacterial concentration was quantified by measuring the optical density (OD) at 600 nm in a Synergy HTX Multi-Mode Reader spectrophotometer (Agilent, Santa Clara, CA, USA). Bacterial concentration was further confirmed by serial dilution and determination of colony-forming units (CFU) on Mueller–Hinton agar plates. *E. coli* 11,501 were grown at 37 °C until the mid-log phase, then centrifuged and washed twice with PBS. Prior to addition to the IPEC-J2 or moDC cells, the bacteria were diluted in a serum and antibiotic-free IPEC-J2 or moDC cell culture medium to a concentration corresponding to a multiplicity of infection (MOI) of 50 bacteria per cell.

### Design of experiments in IPEC-J2 and moDC monocultures

**Fig. 1:**
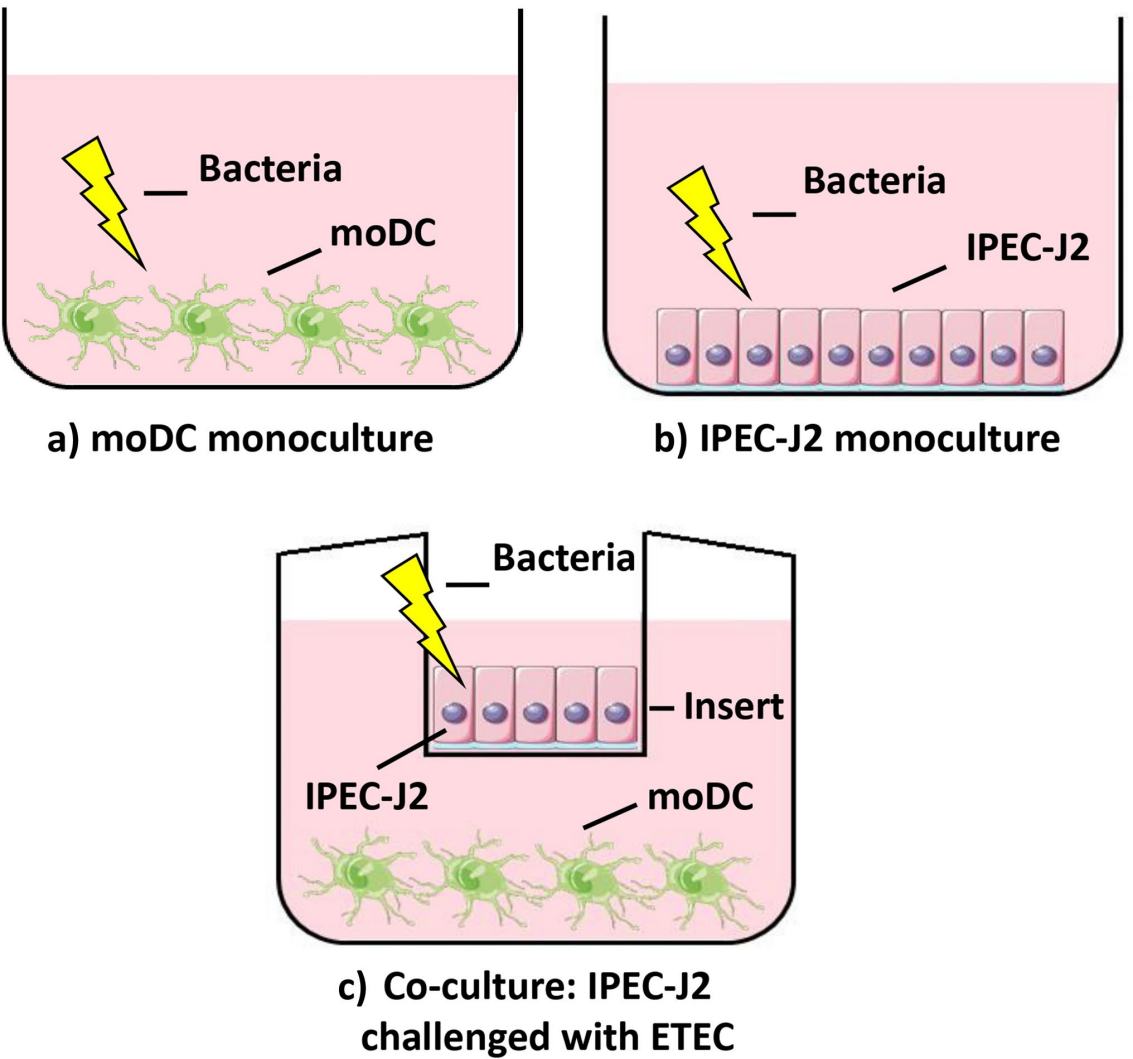
Schematic diagram showing monoculture and co-culture system. **a-b**) IPEC-J2 or moDC monocultures seeded onto 12-well culture plates; **c**) IPEC-J2 cells seeded onto 12-well Transwell^®^-COL membrane inserts at a density of 2.5 × 10^5^ cells/cm^2^ and cultured for 15 days. Porcine moDC seeded onto 12-well plates at a cell density of 4,5 × 10^5^ cells/cm^2^ and cultured for 5 days. IPEC-J2 cell inserts were placed in the wells above the moDC

In the IPEC-J2 + EPS (or moDC + EPS) experimental group, the cells were incubated in DMEM/F12 (or RPMI 1640) medium containing 100 µg/mL EPS-L26 for 4 h (37 °C, 5% CO_2_), while in the experiments testing the effect of ETEC infection (IPEC-J2 + ETEC; moDC + ETEC), the cells were treated with a culture medium containing ETEC (MOI 50:1) and incubated for 2 h (37 °C, 5% CO_2_) (Table 2). In the pretreatment experimental groups, i.e., IPEC-J2 + EPS + ETEC or moDC + EPS + ETEC, cells were incubated with EPS-L26 (100 µg/mL) for 4 h (37 °C, 5% CO_2_) followed by challenge with ETEC (MOI 50:1) for 2 h (37 °C, 5% CO_2_) (Fig. 1a-b). The control group of cells were incubated in DMEM/F12 (or RPMI 1640) medium only without any supplementation.

**Table 2:** Table of cell treatment in monocultures

Type of cell treatment	Experimental design of monocultures	
Control	IPEC-J2 or moDC cells without treatment	-
ETEC infection	IPEC-J2 or moDC cells challenged with ETEC for 2 h	ETEC
EPS-L26 treatment	IPEC-J2 or moDC cells treated with EPS-L26 (100 μg/mL) for 4 h	EPS
EPS-L26 pretreatment	IPEC-J2 or moDC cells treated with EPS-L26 (100 µg/mL) for 4 h and then challenged with ETEC and incubated for another 2 h	EPS + ETEC

### Design of experiments in IPEC-J2/moDC co-cultures

After reaching high TEER values ± 15 days after planting (> 1300 Ω), IPEC-J2 cells were inserted into culture plates with prepared immature moDC cells and co-cultured together for adaptation overnight at 37 °C in a humidified atmosphere of 5% CO_2_ in DMEM/F12 medium without any supplementation. After 24 h of co-cultivation, the cells on the inserts and in the bottom wells were washed twice with PBS. In the IPEC-J2 + EPS experimental group, EPS-L26 (100 µg/mL) was applied to the apical side (IPEC-J2) and DMEM/F12 medium was added to the moDC cells (basolateral side), and the plates were further incubated at 37 °C, 5% CO_2_, for 4 h. In the experimental group testing the effect of ETEC infection (IPEC-J2 + ETEC), culture medium containing ETEC (MOI 50:1) was added to IPEC-J2 cells (apical side) and DMEM/F12 medium was added to the moDC cells (basolateral side) and the plates were incubated for 2 h (37 °C, 5% CO_2_) (Fig. 1c). In the pretreatment experimental group, IPEC-J2 cells (apical side) were treated with EPS-L26 (100 µg/mL) for 4 h (37 °C, 5% CO_2_) and then challenged with ETEC (MOI 50:1) for 2 h (37 °C, 5% CO_2_). The control group of cells (IPEC-J2) remained untreated and incubated in DMEM//F12 medium without any supplements. In the co-culture model, the basolateral side with planted moDC cells was untreated, and incubated in DMEM/F12 medium without any supplements (Table 3). After the indicated incubation period, samples of supernatant (500 µL) were taken from individual wells for subsequent detection of IL-8 and TNF-α secretion, while total RNA was isolated from the cells in the wells to assess the gene expression of chemokines and cytokines using qPCR.

**Table 3:** Table of cell treatment in co-cultures

Type of cell treatment	Experimental design of co-cultures	Abbreviation
Control	IPEC-J2 cells (apical side) and moDC (basolateral side) without treatment	-
ETEC infection	IPEC-J2 cells (apical side) challenged with ETEC for 2 h; moDC (basolateral side) without treatment	ETEC
EPS-L26 treatment	IPEC-J2 cells treated with EPS-L26 (100 μg/mL) for 4 h; moDC (basolateral side) without treatment	EPS
EPS-L26 pretreatment	IPEC-J2 cells treated with EPS-L26 (100 µg/mL) for 4 h and then infected with ETEC and incubated for another 2 h; moDC (basolateral side) without treatment	EPS + ETEC

## Analysis of gene expression

### Isolation of RNA and synthesis of complementary cDNA

Total RNA was isolated using the PureZOL™ reagent (BioRad, USA) exactly according to the manufacturer's instructions. The purity and the concentration of the purified RNA was measured at 260/280 nm, using a NanoDrop 8000 (Thermo Scientific, USA), and the RNA was then transcribed into cDNA using the QuantiTect kit (Qiagen, Germany) exactly according to the manufacturer's instructions. The resulting cDNA was stored at -18 °C until use.

### Quantitative Polymerase Chain Reaction (qPCR)

Gene expression analysis was performed using CFX Manager software (CFX Manager version 2.0, BioRad, USA) on an iCycler CFX 96 (BioRad, USA) in a reaction volume of 10 µL, consisting of the following reagents: 1 × iQ™ SYBR® Green Supermix (BioRad), 0.5 µmol/L forward and reverse primer and 40 µg/µL cDNA (from IPEC-J2 or moDC). The primers used in this study are listed in Table 4. GAPDH was used as a reference gene for internal control in the case of IPEC-J2 cells and β-actin was used as an internal control for moDC. The experimental protocol of qPCR consisted of an initial denaturation at 95 °C for 5 min, followed by amplification in 40 cycles consisting of 3 steps (denaturation at 94 °C for 30 s, hybridization at 60 °C for 30 s, extension at 72 °C for 30 s) and final extension at 72 °C for 15 min followed by melting curve analysis to confirm amplification of the specific product. The relative normalized expression was evaluated using the 2^−∆∆CT^ method. The gene expression analysis was done in triplicates and is expressed as mean ± standard deviation (SD).

**Table 4:** Primers and amplicon length

Gene	Primer name	Sequence 5´ → 3´	Amplicon length	Reference
β-actin	BA/F	CAT CAC CAT CGG CAA CGA	143 bp	Moue et al. 2008
	BA/R	GCG TAG AGG TCC TTC CTG ATG T		
GAPDH	GA/F	ACT CAC TCT TCT ACC TTT GAT GCT	100 bp	Loss et al. 2018
	GA/R	TGT TGC TGT AGC CAA ATT CA		
TNF-α	TNF-α/F	CGA CTC AGT GCC GAG ATC AA	58 bp	Kšonžeková et al. 2016
	TNF-α/R	CCT GCC CAG ATT CAG CAA AG		
IL-1β	IL-1β/F	GCC CTG TAC CCC AAC TGG TA	61 bp	This study
	IL-1β/R	CCA GGA AGA CGG GCT TTT G		
IL-6	IL-6/F	TGG ATA AGC TGC AGT CAC AG	109 bp	Moue et al. 2008
	IL-6/R	ATT ATC CGA ATG GCC CTC AG		
IL-8	IL-8/F	CGC ATT CCA CAC CTT TCC ACC CC	130 bp	This study
	IL-8/R	TCC TTG GGG TCC AGG CAG ACC		
IL-12p35	IL-12p35/F	AGT TCC AGG CCA TGA ATG CA	124 bp	Moue et al. 2008
	IL-12p35/R	TGG CAC AGT CTC ACT GTT GA		
TLR4	TLR4/F	CTC TGC CTT CAC TAC AGA GA	321 bp	Moue et al. 2008
	TLR4/R	CTC TGC CTT CAC TAC AGA GA		
TLR5	TLR5/F	TTT CTG GCA ATG GCT GGA CA	318 bp	Moue et al. 2008
	TLR5/R	TGG AGG TTG TCA AGT CCA TG		
MyD88	MYD88/F	TGA AGC GCA GCA GGA GGC A	160 bp	This study
	MYD88/R	TCG CTG GGG CAG TAG CAG ATG A		
NF-κB	NF-κB/F	GGA GCT GGT GGA GGC CCT GA	152 bp	Kšonžeková et al. 2016
	NF-κB/R	GCC TTG TGG AGG CAG GCG AG		

### Enzyme-Linked Immunosorbent Assay (ELISA)

For the analysis of cytokine release from moDC or IPEC-J2 cells, cell-free cell culture supernatants were collected at the end of the indicated incubation times for each experimental group, centrifuged (300 g for 5 min), and stored at -80 °C until use. IL-8 and TNF-α concentrations were determined by using the following ELISA kits according to the manufacturer’s instructions: porcine IL-8 ELISA (Swine IL-8 ELISA Kit, Thermo Fisher, USA) and porcine TNF-α (Swine TNF-α ELISA Kit, Thermo Fisher, USA). A Synergy HTX microplate reader (Agilent, USA) was used to measure the absorbance values. OD-specific sample concentrations were calculated from a standard curve using a four-parameter logistic curve fit. The results are expressed in pg/mL.

### Statistical analysis

Gene expression results are expressed as the mean and standard deviation (SD) of two independent experiments conducted in triplicates. Significant differences were determined using GraphPad Prism 9.0.0 software by one-way analysis of variance (ANOVA) followed by Dunnett's multiple comparisons. The level of significance was set as P-value ≤ 0.05 considered significant (*), P-value ≤ 0.01 considered highly significant (**), P-value ≤ 0.001 (***) considered highly significant, and ns considered insignificant.

## Results

### Optimization of moDC cultivation

#### Immunophenotype and morphology of moDC

**Fig. 2:**
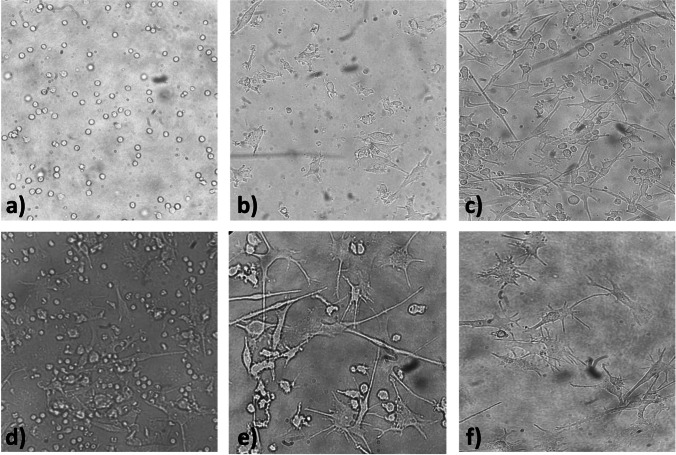
Progression of moDC differentiation by light microscopy (400 x). **a**) Freshly isolated monocytes on day 0 of culture with a characteristic round shape morphology. **b**) The presence of the growth factors IL-4 and GM-CSF in the culture media induces moDC differentiation and maturation. Increased elongation of cytoplasmic processes was observed as early as day 3 of culture. The round cell morphology characteristic of monocytes was lost and the cells acquired an elongated shape. **c**-**d**) Immature moDCs on day 5 of differentiation; **e**–**f**) Fully mature moDCs after 48 h of activation with LPS (100 ng/mL) on day 7 of differentiation

In this study, monocytes were isolated from porcine peripheral blood mononuclear leukocytes (PBMCs) using non-magnetic microbeads labeled with a mouse-specific IgG antibody specific for porcine CD14. After this selective separation, negligible contamination by lymphocytes (up to 10%) was observed in the monocyte suspension. The isolated monocytes were cultured for 5 days in RPMI 1640 medium supplemented with IL-4 and GM-CSF cytokines. During this period, the cell morphology and differentiation were regularly monitored by light microscopy. The shape of the cultured cells on day 0 was round, characteristic for monocytes (Fig. 2a). On day 3 of cultivation, increased cytoplasmic processes were observed as the dendritic cells matured (Fig. 2b). On day 5 of cultivation, a population of moDCs was formed with a subpopulation of adherent and non-adherent cells. The non-adherent cells represented a smaller sub-population of the analyzed cells (approximately up to 15%). The adherent cells showed numerous cytoplasmic processes (Fig. 2c, d).

**Fig. 3:**
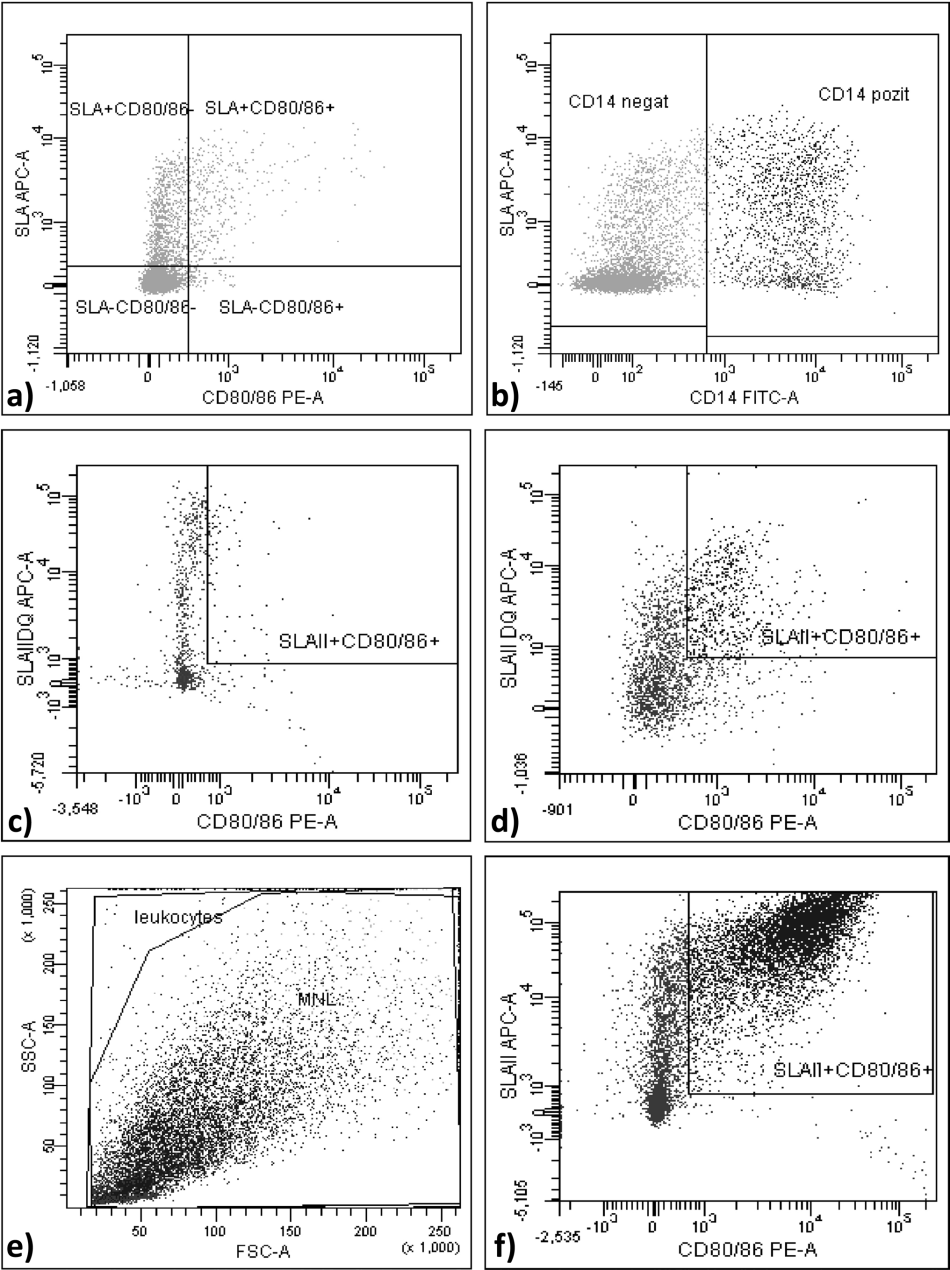
FACS analysis during moDC differentiation. Legend: APC – allophycocyanin, FITC – fluorescein isothiocyanate, PE – phycoerythrin, SLA II – MHC II

Changes in the immunophenotype of the cells were evaluated by flow cytometry. Without prior activation with LPS (day 5), a population of moDCs with the following surface markers was identified: 71% of the analyzed cells showed an immature immunophenotype MHC II^−^ and CD80/86^−^, while 9.6% of the cells showed an immunophenotype MHC II^+^ and CD80/86^+^ (Fig. 3a). Cells were characterized during differentiation by the presence of the cell marker CD14. On day 5 of culture, approximately 30% of the cells analyzed retained the monocyte-specific marker CD14 (Fig. 3b).

Cells were incubated for two days prior to FACS analysis in moDC culture media containing 0.005% hydrocortisone and 0.5% ascorbic acid to prevent pre-activation of the cells. This treatment reduced the immunophenotype with MHC II^+^ and CD80/86^+^ features to 7.9% on day 5 of culture (Fig. 3c). Bacterial LPS (100 ng/mL) was used as a positive control for moDC activation. Cells were treated with LPS for an additional 48 h from day 5, resulting in a significant increase in the expression of MHC II^+^ and CD80/86^+^ maturation markers (47.6%) on day 7 (Fig. 3d-f).

### Optimization of cell co-cultures using the Transwell^®^-COL systemTEER values

The widely accepted quantitative method for assessing the dynamics and integrity of tight junctions (TJs) in 3D in vitro cell models is the measurement of transepithelial electrical resistance (TEER) (Srinivasan et al. 2015).

**Fig. 4:**
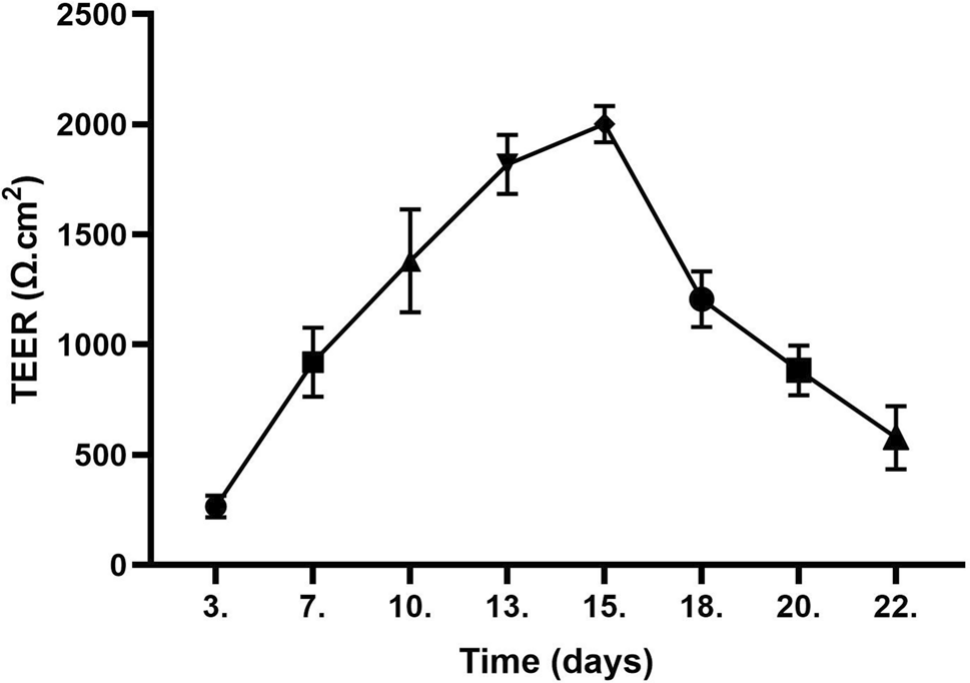
Evaluation of transepithelial electrical resistance (TEER). Mean TEER values of a monolayer of IPEC-J2 cells on Transwell^®^—COL collagen-coated inserts (area 1.12 cm^2^; pore size 0.4 μm, ± SD n = 2) measured over 22 days of cultivation

In this study, a collagen-coated Transwell^®^-COL insert (with a surface area of 1.12 cm^2^ and a pore size of 0.4 μm) was used. IPEC-J2 cells were plated on these inserts and maintained for 22 days. During this period, the cells were regularly examined by light microscopy. The measured electrical resistance of the formed monolayer reached its maximum values on the 15th day of planting (2050 ± 80 Ω x cm^2^). After reaching this maximum, a rapid decrease in TEER values was observed in the following days (Fig. 4).

#### Assessment of paracellular permeability

**Fig. 5:**
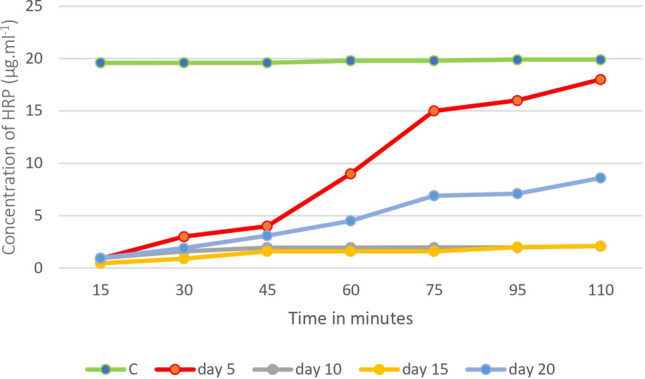
Macromolecular permeability of the IPEC-J2 monolayer formed on collagen-coated Transwell^®^—COL inserts. Basolateral HRP concentration was measured during 110 min incubation. Legend: C – inserts without cells; Day 5–20: inserts with IPEC-J2 cells on a corresponding day after planting

The results of the paracellular permeability test of the IPEC-J2 monolayer on Transwell^®^-COL inserts confirmed the trend of the TEER values measured during the 22-day incubation period. The first measurement of macromolecular permeability was performed on day 5 of culture, when a gradual increase in enzyme transfer to the lower chamber was observed after 45 min of incubation with horseradish peroxidase (HRP) (Fig. 5). By day 10, the tight junctions between the cells were strong enough to prevent any HRP permeation through the IPEC-J2 monolayer, even after 110 min of incubation. This tight junction impermeability was also observed on day 15 when the maximum TEER values were measured. However, the results of the subsequent measurement on day 20 indicated a gradual loosening of the tight junction integrity as the permeability to HRP started to increase after 45 min of incubation (Fig. 5). The observed decreasing TEER values confirm the increasing permeability of the IPEC-J2 monolayer after 20 days of cultivation on the inserts.

##### Transcriptional answer of inflammation-related genes in monocultures

**Fig. 6:**
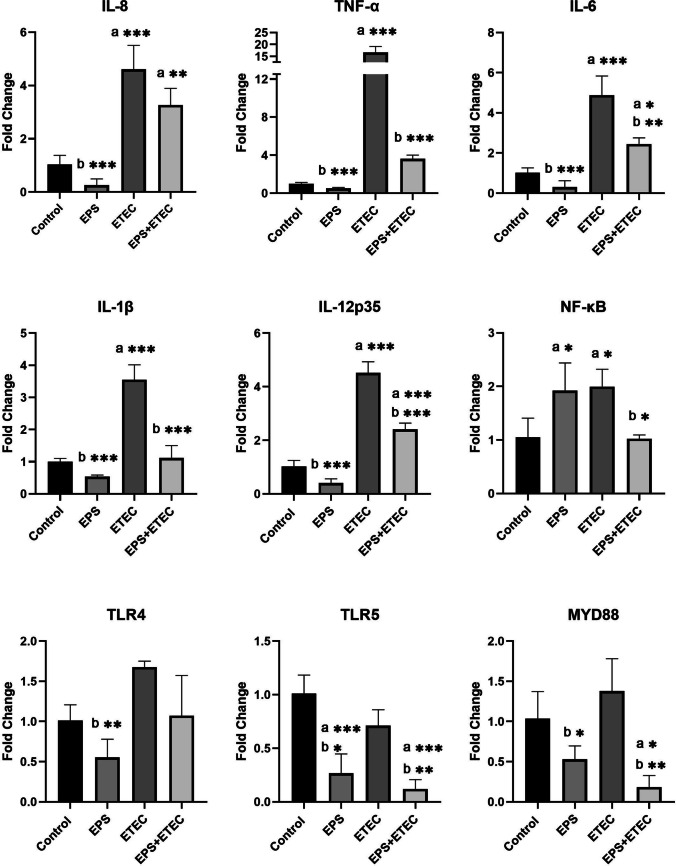
Immunomodulatory activity of EPS-L26 and ETEC in IPEC-J2 monocultures. Results are expressed as mean ± SD). The statistical significance of the differences was evaluated using Dunnett's multiple comparison test. a—Results significantly differed from the control group of cells without treatment (* p < 0.05; ** p < 0.01; *** p < 0.001). b—Results significantly differed from ETEC (* p < 0.05; ** p < 0.01; *** p < 0.001). Error bars represent standard deviations. Legend: Control – cells treated with IPEC-J2 medium (without FBS and antibiotics); EPS – cells treated with EPS-L26 (100 ug/mL) for 4 h; ETEC – cells challenged with ETEC for 2 h; EPS + ETEC – cells treated with EPS-L26 (100 ug/mL) for 4 h followed by 2 h of ETEC challenge

The pro-inflammatory effects of enterotoxigenic *E. coli* (ETEC) were monitored in an in vitro monolayer model of IPEC-J2 and moDC cells separately, as well as in their co-culture model IPEC-J2/moDC. The effect of ETEC was evaluated by gene expression analysis of the transcription factor NF-кB, the intracellular signaling molecule MyD88, pro-inflammatory cytokines (IL-1β, IL-6, IL-8, IL-12p35, TNF-α), and TLR4 and TLR5 receptors. Results obtained from the IPEC-J2 cell model after 2 h of ETEC challenge indicate the ability of these bacteria to induce an inflammatory response compared to the control group of untreated cells (Fig. 6). Stimulation of the cells resulted in increased gene expression of IL-8, IL-6, TNF-α, IL-12p35 and IL-1β (P < 0.001) in infected cells (Fig. 6). A significant increase in mRNA levels of NF-кB was observed (P < 0.05), but the expression of the signaling molecule MyD88 was not statistically significant. Although the gene expression for the TLR4 receptor was increased compared to the control, it was not statistically significant (Fig. 6).

The effect of purified EPS obtained from the probiotic strain *Limosilactobacillus* (formerly *Lactobacillus*) *reuteri* L26 Biocenol™ (EPS-L26) was also analysed on single cell cultures separately and in a co-culture model. Results from gene expression analysis of pro-inflammatory cytokines indicate an immunoregulatory property of EPS-L26 in the experimental IPEC-J2 monolayer model. The mRNA levels of the pro-inflammatory cytokines (IL-1β, IL-8, IL-6, IL-12p35, TNF-α) were not significantly affected in terms of up- or down-regulation compared to a control group of cells, however, a significant down-regulation in the levels of IL-8, TNF-α, IL-6, IL-1β, IL-12p35 (P < 0.001), TLR4 (P < 0.01), MyD88, and TLR5 (P < 0.05) was found compared to the group of ETEC-challenged cells (Fig. 6). EPS also influenced the results obtained from the experimental group of IPEC-J2 cells, which were pretreated with EPS-L26 (100 μg/mL) for 4 h and then challenged with ETEC for 2 h (EPS + ETEC). This pretreatment down-regulated the expression of pro-inflammatory cytokines such as TNF-α, IL-12p35, IL-1β (P < 0.001), and IL-6 (P < 0.01) compared to the group of cells challenged with ETEC alone (Fig. 6). Comparable down-regulation was found for the signaling molecule MyD88 (P < 0.01) and the transcription factor NF-κB (P < 0.05), which are involved in the activation of the TLR4 and TLR5 signaling pathways. In this pretreated group of IPEC-J2 cells, the expression of TLR4 and TLR5 was also suppressed compared to the group of cells only challenged with ETEC alone, but statistical significance was found only for TLR5 (P < 0.01) compared to the ETEC group (Fig. 6).

**Fig. 7:**
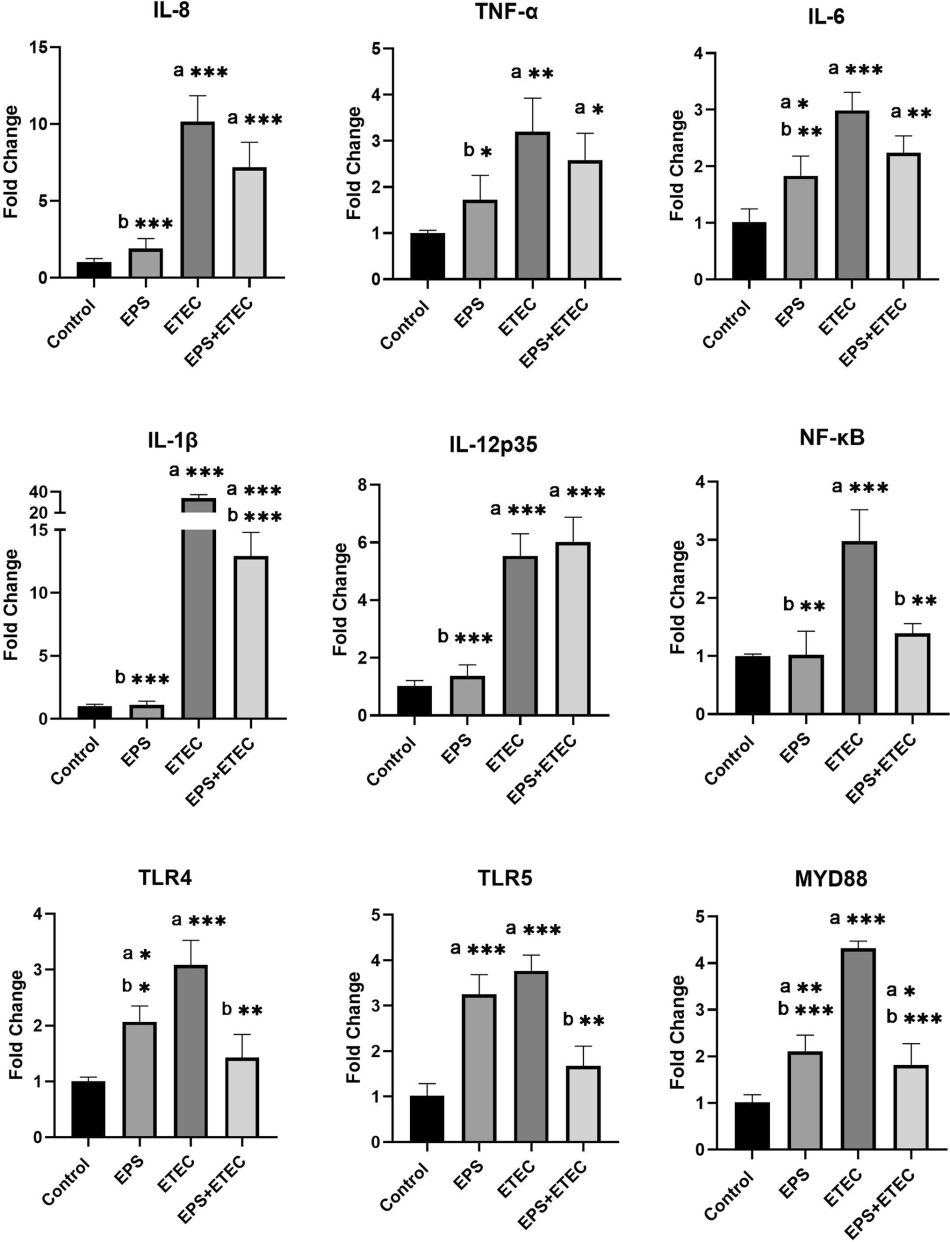
Immunomodulatory activity of EPS-L26 and ETEC in moDC monocultures. Results are expressed as mean ± SD. The statistical significance of the differences was evaluated using Dunnett's multiple comparison test. a—Results significantly different from the control group of untreated cells (* p < 0.05; ** p < 0.01; *** p < 0.001). b—Results significantly different from ETEC (* p < 0.05; ** p < 0.01; *** p < 0.001). Error bars represent standard deviations. Legend: Control – cells treated with moDC medium (without FBS and antibiotics); EPS – cells treated with EPS-L26 (100 ug/mL) for 4 h; ETEC – cells challenged with ETEC for 2 h; EPS + ETEC – cells treated with EPS-L26 (100 ug/mL) for 4 h followed by 2 h of ETEC challenge

The ability of ETEC to induce an inflammatory response was also demonstrated in the moDC monoculture model after 2 h of ETEC challenge when a significant increase in gene expression was observed for IL-8, IL-6, IL-1β, IL-12p35 (P < 0.001) and TNF-α (P < 0.01) compared to the control. The mRNA levels for the transcription factor NF-кB and the signaling molecule MyD88 were also up-regulated (P < 0.001) compared to the control (Fig. 7). Similarly, the gene expression of the TLR4 and TLR5 receptors was also stimulated by ETEC, leading to their up-regulation with a statistical significance of P < 0.001 (Fig. 7).

The effect of purified EPS-L26 (100 μg/mL) on the moDC monolayer was very similar to its effect on the IPEC-J2 cell monolayer. A significant downregulation of the mRNA expression levels of the following cytokines was observed: IL-8, IL-1β, IL-12p35, MyD88, TLR5 (P < 0.001), IL-6, NF-kB (P < 0.01), TNF-α and TLR4 (P < 0.05) compared to the experimental ETEC group. Among the pro-inflammatory cytokines, only the gene expression of IL-6 (P < 0.05) was significantly increased in the EPS-L26-treated experimental moDC group compared to the control (Fig. 7). Pretreatment of moDC cells with EPS-L26 (100 μg/mL) was able to inhibit the increase in gene expression of the investigated pro-inflammatory cytokines induced by ETEC challenge. The expression of genes encoding the cytokine IL-1β was significantly reduced compared to the ETEC group (P < 0.001). Similarly, the expression of genes encoding TLR4 and TLR5 (P < 0.01), the signaling molecule MyD88 (P < 0.001), and the transcription factor NF-кB (P < 0.01) were significantly reduced compared to the ETEC group (Fig. 7).

##### Transcriptional answer of inflammation-related genes in co-cultures

In the co-culture model experiment, the influence of purified EPS-L26 on the immune response of IPEC-J2 cells and the pro-inflammatory effect of ETEC was also investigated. Gene expression levels were assessed by qPCR for pro-inflammatory cytokines (IL-8, IL-6, IL-1β, IL-12p35 TNF-α), the intracellular signaling molecule MyD88, the transcription factor NF-κB, and the genes encoding TLR4 and TLR5 receptors. In this co-culture model, moDC cells located in the basolateral compartment were not treated directly with either EPS-L26 or ETEC.

**Fig. 8:**
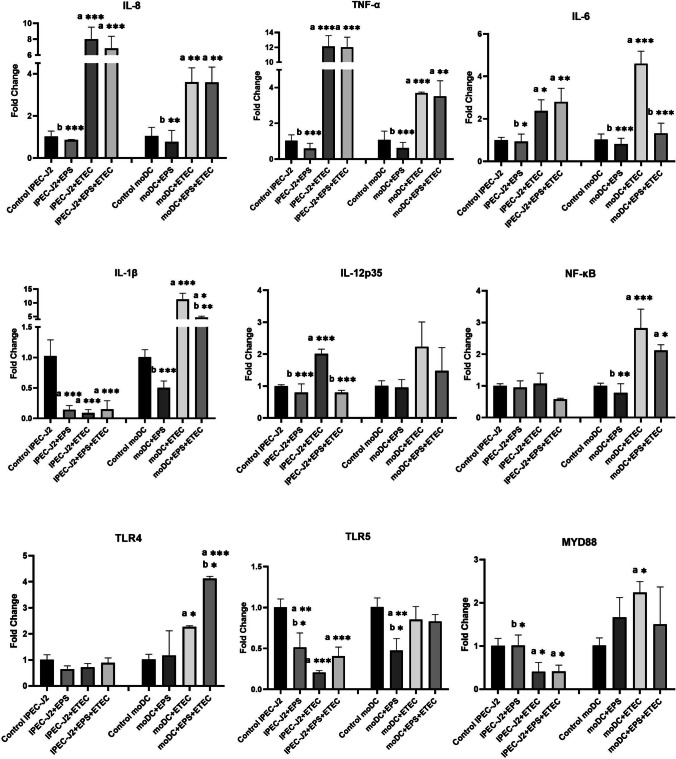
Immunomodulatory activity of EPS-L26 and ETEC in IPEC-J2/moDC co-cultures. Results are expressed as mean ± SD). The statistical significance of differences was evaluated using Dunnett's multiple comparison test. a – Results significantly different from the control group of untreated cells (* p < 0.05; ** p < 0.01; *** p < 0.001) in each cell culture. b—Results significantly different from ETEC (* p < 0.05; ** p < 0.01; *** p < 0.001) in each cell culture. Error bars represent standard deviations. Legend: Control IPEC-J2 – IPEC-J2 cells only; IPEC-J2 + EPS – IPEC-J2 cells treated with EPS-L26 (100 µg/mL) for 4 h; IPEC-J2 + ETEC – IPEC-J2 cells challenged with ETEC for 2 h; IPEC-J2 + EPS + ETEC – IPEC-J2 cells treated with EPS-L26 (100 µg/mL) for 4 h followed by 2 h of ETEC challenge; Control moDC – moDC cells only; moDC + EPS – moDC cells treated with EPS-L26 (100 µg/mL) for 4 h; moDC + ETEC – moDC cells challenged with ETEC for 2 h; moDC + EPS + ETEC – moDC cells treated with EPS-L26 (100 µg/mL) for 4 h followed by 2 h of ETEC challenge

ETEC challenge of IPEC-J2 cells on inserts (apical side) resulted in increased gene expression of IL-8, IL-12p35, TNF-α (P < 0.001), and IL-6 (P < 0.05) compared to the control group of IPEC-J2 cells. Similarly, increased gene expression for IL-6, TNF-α, IL-1β, NF-κB (P < 0.001), and IL-8 (P < 0.01), MyD88, and TLR4 (P < 0.05) was observed in directly uninfected moDC cells (moDC + ETEC) (Fig. 8).

The effect of purified EPS-L26 applied to the apical side of the insert membranes was manifested in IPEC-J2 by down-regulation of gene expression for IL-1β (P < 0.001) and TLR5 (P < 0.01) compared to the control group. In parallel, there was a downregulation of gene expression for IL-8, TNF-α, IL-12p35 (P < 0.001), and IL-6 (P < 0.05) in this experimental group compared to cells challenged with ETEC. Very similar results were obtained from directly untreated moDC cells, where a reduction in gene expression was observed for the following cytokines IL-6, TNF-α, IL-1β (P < 0.001), IL-8, NF-κB (P < 0.01), and TLR5 (P < 0.05) compared to indirect ETEC infection in the experimental moDC + ETEC group (Fig. 8).

Pretreatment of IPEC-J2 cells (on insert membranes) followed by a 2 h infection with ETEC resulted in decreased mRNA levels for IL-1β, TLR5 (P < 0.001), and MyD88 (P < 0.05), but increased mRNA levels for IL-8, TNF-α (P < 0.001), and IL-6 (P < 0.01) compared to the control group of IPEC-J2 cells. Similar results were obtained for moDC cells that were not directly treated, but showed increased statistical significance compared to the control group for the following cytokines: IL-8, TNF-α, TLR4 (P < 0.01), IL-1β, and NF-κB (P < 0.05) (Fig. 8). The putative immunosuppressive effect of EPS-L26 applied to IPEC-J2 cells on insert membranes was observed in the moDC cell culture, where down-regulation of cytokines IL-6 (P < 0.001) and IL-1β (P < 0.01) was detected compared to indirect ETEC infection in the experimental moDC + ETEC group. The immunosuppressive potential of EPS-L26 was also observed in IPEC-J2 cells seeded on semi-permeable Transwell^®^ inserts in the case of the gene encoding the cytokine IL-12p35, which was down-regulated compared to ETEC infection of IPEC-J2 cells (P < 0.001) (Fig. 8).

#### Secretion of IL-8 and TNF-α

**Fig. 9:**
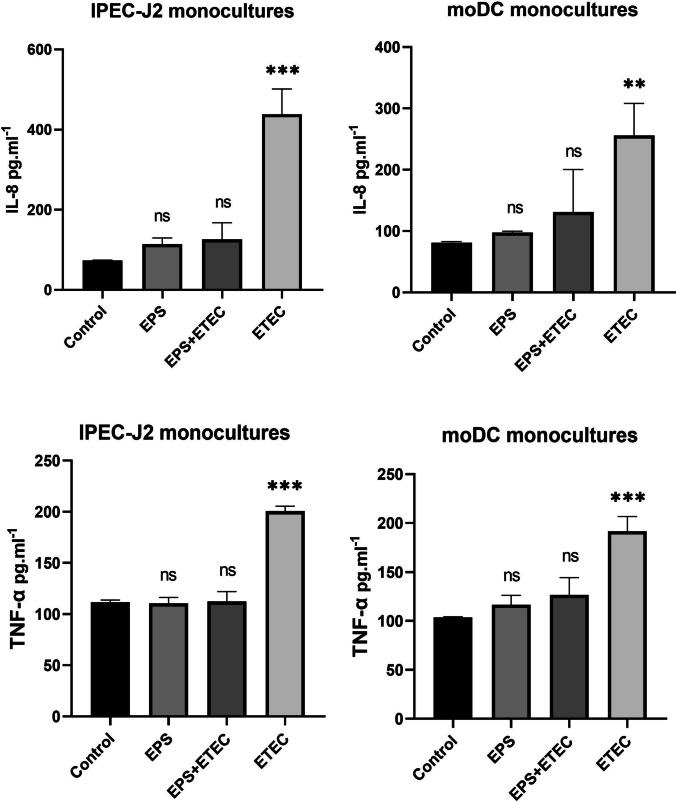
Secretion of IL-8 and TNF-α in the cell supernatant of IPEC-J2 and moDC monocultures. Results are expressed as mean ± SD. The statistical significance of differences was evaluated using Dunnett's multiple comparison test. Results were significantly different from the control group of untreated cells (* p < 0.05; ** p < 0.01; *** p < 0.001). Error bars represent standard deviations. Legend: Control – IPEC-J2 cells, or moDCs only; EPS – IPEC-J2, or moDCs treated with EPS-L26 (100 µg/mL) for 4 h; ETEC – IPEC-J2, or moDCs challenged with ETEC for 2 h; EPS + ETEC – IPEC-J2, or moDCs treated with EPS-L26 (100 µg/mL) for 4 h followed by 2 h of ETEC challenge

In supernatants of monocultured IPEC-J2 cells, the ETEC challenge for 2 h resulted in the highest level release of IL-8 secretion compared to the control group of IPEC-J2 cells (435 ± 40 pg/mL; P < 0.001). Identical results were obtained in cell supernatants of monocultured moDCs, where the highest secretion of IL-8 was also observed in moDCs cells challenged with ETEC (265 ± 35 pg/mL; P < 0.01) (Fig. 9).

Analysis of TNF-α levels in cell supernatants from ETEC-challenged IPEC-J2 monocultures showed a significantly increased concentration compared to the control (200 ± 3 pg/mL; P < 0.001), and the same significant increase in TNF-α concentration (192 ± 15 pg/mL; P < 0.001) was observed in supernatants from ETEC-challenged moDC monocultures. The secretion of IL-8 and TNF-α into the supernatants of IPEC-J2 cells and moDC cells treated with EPS-L26 alone (100 ug/mL), and cells pretreated with EPS-L26 and then challenged with ETEC for 2 h, was not significantly different from the control (Fig. 9).

**Fig. 10:**
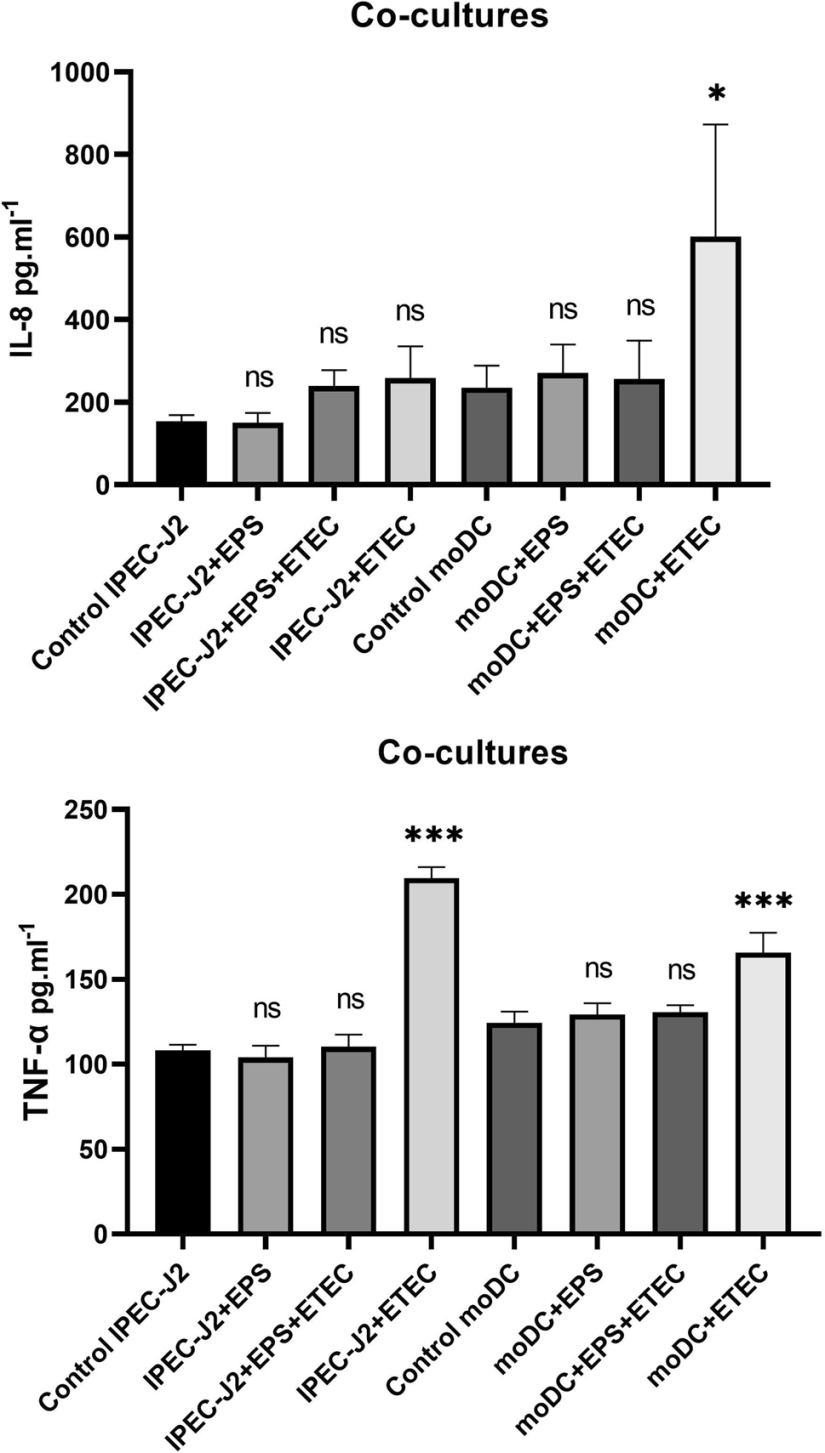
Secretion of IL-8 and TNF-α into the cell supernatant in IPEC-J2/moDC co-cultures. Results are expressed as mean ± SD. The statistical significance of differences was evaluated using Dunnett's multiple comparison test. Results were significantly different from the control group of untreated cells (* p < 0.05; ** p < 0.01; *** p < 0.001) in the respective cell culture. Error bars represent standard deviations. Legend: Control IPEC-J2 – IPEC-J2 cells only; IPEC-J2 + EPS – IPEC-J2 cells treated with EPS-L26 (100 µg/mL) for 4 h; IPEC-J2 + ETEC – IPEC-J2 cells challenged with ETEC for 2 h; IPEC-J2 + EPS + ETEC – IPEC-J2 cells treated with EPS-L26 (100 µg/mL) for 4 h followed by 2 h of ETEC challenge; Control moDC – moDC cells only; moDC + EPS – moDC cells treated with EPS-L26 (100 µg/mL) for 4 h; moDC + ETEC – moDC cells challenged with ETEC for 2 h; moDC + EPS + ETEC – moDC cells treated with EPS-L26 (100 µg/mL) for 4 h followed by 2 h of ETEC challenge

In the co-culture cell model, where communication between two co-cultured cell monocultures is assumed, increased secretion of IL-8 into the cell supernatant was observed in the case of ETEC challenge, i.e. the moDC + ETEC experimental group (585 ± 190 pg/mL; P < 0.05), in the supernatants of indirectly challenged moDCs, and also for TNF-α (165 ± 10 pg/mL) (Fig. 10). In this cell model, we also found an increased release of TNF-α (212 ± 5 pg/mL) in the supernatants of IPEC-J2 cells directly challenged with ETEC, with a statistical significance of P < 0.001 compared to the control group of untreated IPEC-J2 cells (Fig. 10).

## Discussion

It is well known that dendritic cells (DCs) are among the major antigen-presenting cells (APCs) found in the lung mucosa, skin epithelia, and intestinal *lamina propria* and play a key role in antigen capture. In tissues, DCs are strategically located in areas with the highest risk of pathogen entry and immediately interact with pathogen-associated molecular patterns (PAMPs), leading to their activation (Shin et al. 2019).

The low number of conventional DCs in the bloodstream is the main reason why these cells are obtained by in vitro cultivation of monocytes in a medium enriched with growth factors such as GM-CSF and IL-4 (Singleton et al. 2016).

Despite the complexity of isolation techniques, we were able to obtain a large number of mononuclear cells from porcine peripheral blood, from which we selectively isolated monocytes. On day 5 of culture an immatureimmunophenotype of the moDCs was obtained however after 48 h of cultivation with LPS from *E. coli* O111:B4, we observed an increase in surface marker expression for the MHC class II, CD80/86 surface marker (> 45%). Similar results were obtained other available studies which investigated the ability of live pathogens or their PAMPs to induce the maturation and activation of monocyte-derived macrophages and dendritic cells, with similar results (Carrasco et al. 2001; Kim et al. 2019; Meital 2019).

Exopolysaccharides (EPSs) produced by probiotic bacteria contribute to the immunomodulation of the innate immune response by interacting with pattern recognition receptors (PRRs) such as Toll-like receptors (TLRs) or C-type lectin receptors (CLRs) found on intestinal epithelial cells and immune cells in the *lamina propria* (Oerlemans et al. 2021).

TLRs can recognize EPS molecules and initiate the TLR signaling pathway or, conversely, block their activation through mechanisms of negative regulation. Binding of a specific ligand to the appropriate TLR receptor initiates its dimerization, which subsequently recruits adapter molecules such as MyD88, TIRAP (Mal), or TRIF, leading to the activation of the transcription factors NF-κB, AP-1, or IRF and subsequent up-regulation of inflammatory cytokines, chemokines, various antimicrobial molecules, or co-stimulatory molecules (Yu and Gao 2015).

An interesting finding was that whereas, in our previous study in which IPEC-J2 cells were challenged with *Salmonella* Typhimurium, we observed an up-regulation of genes encoding receptors for TLR4 and TLR5 (Kiššová et al. 2022), in our recent work we did not observe increased gene expression for these receptors in IPEC-J2 monocultures challenged with ETEC. However, a significant up-regulation of genes encoding the transcription factor NF-κB (P < 0.05) and MyD88 (not significant) was observed in the ETEC experimental group compared to the control. Based on our findings, we hypothesize that the activation of transcription factors and subsequent expression of pro-inflammatory cytokines in our study was triggered by the activation of other PRR receptors. On the other hand, in moDC monocultures in the ETEC experimental group, we found increased mRNA levels for TLR4 and TLR5 receptors (P < 0.001) compared to the control group of cells. In this experimental group of ETEC-challenged cells, we also observed significantly increased expression (P < 0.001) for genes encoding NF-κB, and MyD88. Our results suggest that similar to other studies (Lin et al. 2011; Yahya et al. 2019), bacterial EPSs are able to affect lectin receptors and the TLR signaling pathways.

When monitoring the TLR signaling pathway in the IPEC-J2/moDC co-culture model, we found that the expression levels of the cytokines related to the TLR signaling pathway (TLR4, NF-κB, MyD88) were significantly lower in indirectly treated moDCs (basolateral compartment; moDC + ETEC) than in moDC monocultures. Gene expression for genes encoding TLR5 receptors was also down-regulated compared to moDC monocultures. Treatment of IPEC-J2 cells on semi-permeable inserts (apical compartment) led to changes in gene expression of the monitored cytokines in the untreated moDCs located in the lower chamber (basolateral compartment), indicating intercellular signalization between the two co-cultured cell cultures. Similarly, the study by Zhang et al. (2017) confirmed the immunosuppressive properties of anthocyanins derived from purple carrots and potatoes by co-culturing epithelial Caco-2 cells with THP-1 cells. Pretreatment of Caco-2 cells in the apical compartment led to down-regulation of the pro-inflammatory cytokines IL-8, IL-6, IL-1β, and TNF-α in both cell lines, including THP-1 cells cultured in the button culture well, which were not directly treated with anthocyanins (Zhang et al. 2017).

Measurement of transepithelial electrical resistance (TEER) is a standard technique for assessing the barrier integrity of monolayers of in vitro epithelial cell models using insert chambers. Cellular experiments with a confluent monolayer of IPEC-J2 are typically performed at TEER values of 1,000—3,000 Ω × cm^2^. These values are achieved after 4—9 days of cultivation on a semipermeable membrane insert (with a pore size of 0.4 μm) coated or uncoated with a collagen (Vergauwen et al. 2015; Bernardini et al. 2021). The results of our study are consistent with previous findings, as using semipermeable collagen-coated Transwell^®^ inserts cultured in 10% FBS, we achieved maximum TEER values of 2050 ± 80 Ω × cm^2^ on day 15 of cultivation. Prolonged cultivation of IPEC-J2 cells resulted in a gradual loosening of the tight junctions, confirmed by decreasing TEER values and increasing macromolecular permeability of the monolayer to HRP. Similarly, Geens and Niewold (2011), in their study using the IPEC-J2 cell model cultured on Transwell^®^-COL inserts (1.12 cm^2^), confirmed that the cells differentiated into a tight epithelium impermeable to HRP after 10 days of culture, which was further confirmed by the measured increased TEER values (Geens and Niewold 2011).

Pro-inflammatory cytokines released by immune and epithelial cells during ongoing inflammation help to neutralize invaders in the body, but their excessive and prolonged action can have a pathological effect on healthy tissue (Strober and Fuss 2011).

In the present study, we focused on evaluating the gene expression of pro-inflammatory cytokines (IL-8, TNF-α, IL-6, IL-1β, IL-12p35) analyzed by qPCR. The results obtained from the analysis of gene expression in the experimental group of IPEC-J2 monoculture challenged with ETEC were consistent with other studies (Sargeant et al. 2011; Zhou et al. 2012; Yu et al. 2018). We observed a significant increase in gene expression for the pro-inflammatory cytokines IL-8, TNF-α, IL-6, IL-1β, and IL-12p35 (P < 0.001) in the experimental group of IPEC-J2 cells challenged with ETEC compared to the control. The results obtained from the moDC monoculture in the moDC + ETEC experimental group were consistent with the above results obtained from IPEC-J2 + ETEC. mRNA levels of the pro-inflammatory cytokines IL-8, IL-6, IL-1β, IL-12p35 (P < 0.001), and TNF-α (P < 0.01) were significantly up-regulated compared to the control group of moDC cells. Comparably, Devriendt et al. (2010), using purified F4 fimbriae obtained from *E. coli* strain IMM01 to challenge the moDC model, not only achieved maturation of immature moDCs, but also found increased expression of the pro-inflammatory cytokines IL-1β, IL-6, TNF-α, IL-8, and IL-12p40 in mature cells compared to the immature moDC population (Devriendt et al. 2010).

On the contrary, results from the treatment of IPEC-J2 cells with EPS-L26 for 4 h in monoculture suggest an immunosuppressive effect of EPS-L26 on these cells. None of the evaluated mRNA levels of pro-inflammatory cytokines were up-regulated, whereas IL-8, TNF-α, IL-6, IL-1β, and IL-12p35 were down-regulated compared to ETEC-challenged cells (P < 0.001). From the available publications, it is known how the physicochemical properties of EPS molecules influence the immune response of cells in in vitro models, leading to either immunostimulation or immunosuppression. Nishimura-Uemura et al. (2003) using the mouse macrophage cell line J774.1 in the presence of neutral polysaccharide (NPS) or acidic phosphopolysaccharide (APS) from *Lactobacillus delbrueckii* ssp. *bulgaricus* OLL1073R-1, found that treatment of macrophages with APS caused significant morphological changes in the cells, accompanied by up-regulation of gene expression for the cytokine IL-1α. Conversely, macrophages stimulated with NPS showed almost no changes in cell morphology or up-regulated IL-1α gene expression (Nishimura-Uemura et al. 2003). Bleau et al. (2010) showed how the molecular weight of EPS from *Lactobacillus rhamnosus* RW-9595 M affects cytokine production in lymphocytes isolated from mouse spleen. They found that the high molecular weight EPS (> 5 × 10^5^ Da) reduced the production of the pro-inflammatory cytokine IL-6 in cell supernatants compared to the positive control, as the molecular weight of EPS decreased, the release of IL-6 into the supernatants increased (Bleau et al. 2010). Our results are consistent with these studies, as the EPS-L26 used in our study with a molecular weight of 8.2 × 10^5^ Da (Kšonžeková et al. 2016) influenced the treated IPEC-J2 and moDC cells towards immunosuppression.

In a co-culture model, we observed increased gene expression of IL-8, TNF-α, IL-12p35 (P < 0.001), and IL-6 (P < 0.05) in IPEC-J2 cells (apical compartment) directly challenged with ETEC compared to the control group. An interesting finding was that although moDC cells (basolateral compartment) were not directly challenged, we also obtained a significant increase in the mRNA levels of the pro-inflammatory cytokines IL-6, TNF-α, IL-1β (P < 0.001), and IL-8 (P < 0.01) compared to the control group of moDCs. From these results, we concluded that the modulation of immune cells located in the basolateral compartment is mediated by humoral signals derived from IPEC-J2 cells located in the apical compartment, which are capable of crossing the semipermeable membrane. In line with this idea, and suggesting that soluble factors are likely to be responsible for regulating the responses of DCs seeded in the lower culture wells, Rimoldi et al. (2005) showed that human DCs conditioned with intestinal epithelial cell supernatants showed reduced IL-1β secretion after infection with *S*. Typhimurium SL1344 (Rimoldi et al. 2005).

When comparing the gene expression levels of pro-inflammatory cytokines in monocultures directly challenged with ETEC, these increases were more moderate in the co-culture model. For example, in the co-culture model, we observed a down-regulation for the cytokine IL-1β (P < 0.001) and the transcription factor NF-κB (P < 0.05) in the experimental group of IPEC-J2 + ETEC compared to the control, whereas in the monoculture model of IPEC-J2, the fold change for these molecules was significantly increased. An approximately 50% decrease in the relative gene expression levels encoding the cytokines IL-12p35 and IL-6 was detected in the co-culture model of IPEC-J2 cells challenged with ETEC compared to IPEC-J2 monoculture.

Our findings are consistent with those of other studies investigating cell co-cultivation systems. Loss et al. (2018) investigated the beneficial effect of the probiotic strain *Enterococcus faecium* NCIMB 10415 and the pathological effect of ETEC in cell monocultures of IPEC-J2 and moDC, and in IPEC-J2/moDC co-cultures using Transwell^®^ inserts. Their research showed that moDCs from IPEC-J2/moDC co-cultures responded more mildly to ETEC infection than monocultured moDCs; the expression of IL-1β, IL-18, and NLRP3 was attenuated at the mRNA level, and a similar trend was observed with IL-1β protein detected by ELISA (Loss et al. 2018). Paszti-Gere et al. (2014) used a porcine enterohepatic co-culture system to study the effects of LPS on the gene expression profile of the pro-inflammatory cytokines IL-8, TNF-α and CYP enzymes (CYP1A1, CYP1A2, CYP3A29). They found that the LPS-induced changes in hepatocyte monocultures were significantly reduced in the presence of enterocytes (Paszti-Gere et al. 2014). These studies highlight the attenuation of the inflammatory response in infected cells during the co-cultivation of two cell lines, based on the assumption of their mutual interaction through cell-mediated factors and communication between cells.

In this study, we observed that pretreatment of IPEC-J2 cells with EPS-L26 for 4 h modulated of the inflammatory response induced by pathogenic ETEC in the monoculture model. The measured mRNA levels of the cytokines IL-6 (P < 0.01), TNF-α, IL-1β, and IL-12p35 (P < 0.001) were significantly reduced compared to the IPEC-J2 + ETEC experimental group. Similarly, in moDC monocultures, pretreatment of these cells with EPS-L26 for 4 h resulted in decreased expression of genes encoding the pro-inflammatory cytokine IL-1β (P < 0.001) compared to the moDC + ETEC group. Results obtained from the co-culture model, where IPEC-J2 cells were directly pretreated with EPS-L26 on insert chambers, also indicated an immunosuppressive effect of EPS-L26 on moDCs growing in the lower basolateral compartment. In these moDCs, we observed a decrease in the mRNA levels of genes encoding NF-κB (P < 0.05), IL-6, and IL-1β (P < 0.01) compared to the group of indirectly treated moDC + ETEC cells. The immunosuppressive potential of EPS-L26 after 4 h of pretreatment of IPEC-J2 cells cultured on insert chambers was manifested by down-regulation in the case of the gene encoding the cytokine IL-12p35 (P < 0.001) compared to IPEC-J2 + ETEC cells. The obtained results are consistent with our previous study (Tkáčiková et al. 2020), where pretreatment of IPEC-1 cells with EPS-L26 for 4 h attenuated the expression of genes (ADCYAP1, PRKAR1A, CAMK2G) associated with excessive electrolyte loss during ETEC infection activated by LT or ST toxins. In addition, pretreatment of IPEC-1 cells with EPS-L26 reduced the expression of genes encoding proteases compared to ETEC infection. In another of our studies, Kšonžeková et al. (2016) observed inhibition of ETEC adhesion to epithelial cells and subsequent reduction of gene expression of pro-inflammatory cytokines IL-1β and IL-6 induced by ETEC infection after pretreatment of IPEC-1 cells with EPS-L26 (Kšonžeková et al. 2016).

To validate the results obtained from gene expression using qPCR, we decided to analyze the release of cytokines from IPEC-J2 cells into cell culture supernatants in each experimental group. The levels of IL-8 and TNF-α protein secretion into the cell culture supernatants were quantified using ELISA assays. Overall, the highest levels of secreted cytokines IL-8 and TNF-α were observed in the ETEC challenge experimental group with respect to IPEC-J2 and moDC monoculture. The results are also consistent with the changes in gene expression of IL-8 and TNF-α observed in IPEC-J2 and moDC cells in the co-culture model of experimental cell groups challenged with ETEC. Several studies using co-culture models of two different cell populations have consistently demonstrated increased levels of pro-inflammatory cytokines at the mRNA level, as well as increased levels of secreted proteins detected in cell culture supernatants were demonstrated (Paszti-Gere et al. 2014; Gayer et al. 2022).

## Conclusion

In the present study, we created a model that mimics the intestine by co-cultivating IPEC-J2 and moDC cells on Transwell^®^ semipermeable inserts. The aim of the study was to investigate the immunomodulatory potential of the probiotic exopolysaccharide EPS-L26 on the inflammatory response of this in vivo-like model to enterotoxigenic *E. coli*. We demonstrated the immunoprotective effect of EPS-L26, which was able to suppress the enhanced inflammatory response of cells to ETEC infection in the pretreated experimental model. In addition, we demonstrated the ability of the two co-cultured cell cultures to communicate effectively by observing intercellular signaling as manifested by changes in gene expression in directly untreated moDC cells. An important finding was that each cell culture in the co-culture model responded more moderately to ETEC infection than in the monoculture model. These results suggest that the inflammatory response of intestinal epithelial cells is dependent on the cytokine response of immune cells in the *lamina propria* and vice versa. Further research is needed in this regard to identify and study the soluble factors involved in IEC/DC interactions. The use of in vitro co-culture has been shown to be a reliable experimental model for studying host–pathogen interactions.

## Data Availability

Not applicable.

## References

[CR1] Allaire JM, Crowley SM, Law HT, Chang S-Y, Ko H-J, Vallance BA (2018). The Intestinal Epithelium: Central Coordinator of Mucosal Immunity. Trends Immunol.

[CR2] Bernardini C, Algieri C, Mantia LD, Zannoni A, Salaroli R, Trombetti F (2021). Relationship between serum concentration, functional parameters and cell bioenergetics in IPEC-J2 cell line. Histochem Cell Biol.

[CR3] Bleau C, Monges A, Rashidan K, Laverdure J-P, Lacroix M, Van Calsteren M-R (2010). Intermediate chains of exopolysaccharides from Lactobacillus rhamnosus RW-9595M increase IL-10 production by macrophages. J Appl Microbiol.

[CR4] Carrasco CP, Rigden RC, Schaffner R, Gerber H, Neuhaus V, Inumaru S (2001). Porcine dendritic cells generated in vitro: Morphological, phenotypic and functional properties. Immunology.

[CR5] Castro-Bravo N, Wells JM, Margolles A, Ruas-Madiedo P (2018). Interactions of surface exopolysaccharides from bifidobacterium and lactobacillus within the intestinal environment. Front Microbiol.

[CR6] Devriendt B, Verdonck F, Summerfield A, Goddeeris BM, Cox E (2010). Targeting of Escherichia coli F4 fimbriae to Fcγ receptors enhances the maturation of porcine dendritic cells. Vet Immunol Immunopathol.

[CR7] Fairbrother JM, Nadeau É (2019) Colibacillosis: Diseases of swine. John Wiley & Sons, Inc., New Jersey pp 807–834

[CR8] Gayer FA, Fichtner A, Legler TJ, Reichardt HM (2022) A coculture model mimicking the tumor microenvironment unveils mutual interactions between immune cell subtypes and the human seminoma cell line TCam-2. Cells 11:885. 10.3390/cells11050885 10.3390/cells11050885 PMC8909655 35269507

[CR9] Geens MM, Niewold TA (2011). Optimizing culture conditions of a porcine epithelial cell line IPEC-J2 through a histological and physiological characterization. Cytotechnology.

[CR10] Kim SE, Hwang JH, Kim YK, Lee HT (2019). Heterogeneity of porcine bone marrow-derived dendritic cells induced by GM-CSF. PLoS ONE.

[CR11] Kiššová Z, Tkáčiková Ľ, Mudroňová D & Bhide M (2022) Immunomodulatory effect of Lactobacillus reuteri (Limosilactobacillus reuteri) and its exopolysaccharides investigated on epithelial cell line IPEC-J2 challenged with Salmonella Typhimurium. Life 12:1955.10.3390/life12121955 10.3390/life12121955 PMC9788328 36556320

[CR12] Kšonžeková P, Bystrický P, Vlčková S, Pätoprstý V, Pulzová L, Mudroňová D (2016). Exopolysaccharides of Lactobacillus reuteri: Their influence on adherence of E. coli to epithelial cells and inflammatory response. Carbohydr Polym.

[CR13] Kumar SB, Arnipalli SR, Ziouzenkova O (2020). Antibiotics in food chain: The consequences for antibiotic resistance. Antibiotics.

[CR14] Lin M-H, Yang Y-L, Chen Y-P, Hua K-F, Lu C-P, Sheu F (2011). A novel exopolysaccharide from the biofilm of thermus aquaticus YT-1 induces the immune response through Toll-like receptor 2. J Biol Chem.

[CR15] Loss H, Aschenbach JR, Tedin K, Ebner F, Lodemann U (2018). The inflammatory response to enterotoxigenic E. Coli and Probiotic E. Faecium in a Coculture Model of Porcine Intestinal Epithelial and Dendritic Cells. Mediat Inflamm.

[CR16] Meital LA (2019). Simple and effective method for the isolation and culture of human monocytes from small volumes of peripheral blood. J Immunol Methods.

[CR17] Moue M, Tohno M, Shimazu T, Kido T, Aso H, Saito T, Kitazawa H (2008). Toll-like receptor 4 and cytokine expression involved in functional immune response in an originally established porcine intestinal epitheliocyte cell line. Biochim Biophys Acta - Gen Subj.

[CR18] Nishimura-Uemura J, Kitazawa H, Kawai Y, Itoh T, Oda M, Saito T (2003). Functional alteration of murine macrophages stimulated with extracellular polysaccharides from Lactobacillus delbrueckii ssp. bulgaricus OLL1073R-1. Food Microbiol.

[CR19] Oerlemans MMP, Akkerman R, Ferrari M, Walvoort MTC, de Vos P (2021). Benefits of bacteria-derived exopolysaccharides on gastrointestinal microbiota, immunity and health. J Funct Foods.

[CR20] Paszti-Gere E, Matis G, Farkas O, Kulcsar A, Palocz O, Csiko G (2014). The effects of intestinal LPS exposure on inflammatory responses in a porcine enterohepatic co-culture system. Inflammation.

[CR21] Pérez-Ramos A, Nácher-Vázquez M, Notararigo S, López P, Mohedano ML (2016) Current and Future Applications of Bacterial Extracellular Polysaccharides. Probiotics, Prebiotics, and Synbiotics: Bioactive Foods in Health Promotion, Elsevier Inc., Oxford. 10.1016/B978-0-12-802189-7.00022-8

[CR22] Rimoldi M, Chieppa M, Salucci V, Avogadri F, Sonzogni A, Sampietro GM (2005). Intestinal immune homeostasis is regulated by the crosstalk between epithelial cells and dendritic cells. Immunology.

[CR23] Sargeant HR, Miller HM, Shaw MA (2011). Inflammatory response of porcine epithelial IPEC J2 cells to enterotoxigenic E. coli infection is modulated by zinc supplementation. Mol Immunol.

[CR24] Shin K-S, Jeon I, Kim B-S, Kim I-K (2019). Monocyte-derived dendritic cells dictate the memory differentiation of CD8^+^ T cells during acute infection. Front Immunol.

[CR25] Singleton H, Graham SP, Bodman-Smith KB, Frossard JP, Steinbach F (2016). Establishing porcine monocyte-derived macrophage and dendritic cell systems for studying the interaction with PRRSV-1. Front Microbiol.

[CR26] Srinivasan B, Kolli AR, Esch MB, Abaci HE, Shuler ML, Hickman JJ (2015). TEER measurement techniques for in vitro barrier model systems. J Lab Autom.

[CR27] Strober W, Fuss IJ (2011). Proinflammatory cytokines in the pathogenesis of inflammatory bowel diseases. Gastroenterology.

[CR28] Tkáčiková Ľ, Mochnáčová E, Tyagi P, Kiššová Z, Bhide M (2020). Comprehensive mapping of the cell response to E. coli infection in porcine intestinal epithelial cells pretreated with exopolysaccharide derived from Lactobacillus reuteri. Vet Res.

[CR29] Vergauwen H, Tambuyzer B, Jennes K, Degroote J, Wang W, De Smet S (2015). Trolox and ascorbic acid reduce direct and indirect oxidative stress in the IPEC-J2 cells, an In Vitro model for the porcine gastrointestinal tract. PLoS ONE.

[CR30] Xu C, Peng K, She Y, Fu F, Shi Q, Lin Y, Xu C (2023). Preparation of novel trivalent vaccine against enterotoxigenic Escherichia coli for preventing newborn piglet diarrhea. Am J Vet Res.

[CR31] Yahya SMM, Abdelnasser SM, Hamed AR, El Sayed OH, Asker MS (2019). Newly isolated marine bacterial exopolysaccharides enhance antitumor activity in HepG2 cells via affecting key apoptotic factors and activating toll like receptors. Mol Biol Rep.

[CR32] Yu H, Ding X, Shang L, Zeng X, Liu H, Li N (2018). Protective ability of biogenic antimicrobial peptide microcin J25 against enterotoxigenic escherichia coli-induced intestinal epithelial dysfunction and inflammatory responses IPEC-J2 Cells. Front Cell Infect Microbiol.

[CR33] Yu S, Gao N (2015). Compartmentalizing intestinal epithelial cell Toll-like receptors for immune surveillance. Cell Mol Life Sci.

[CR34] Zeuthen LH, Fink LN, Frokiaer H (2008). Epithelial cells prime the immune response to an array of gut-derived commensals towards a tolerogenic phenotype through distinct actions of thymic stromal lymphopoietin and transforming growth factor-β. Immunology.

[CR35] Zhang H, Hassan YI, Renaud J, Liu R, Yang C, Sun Y, Tsao R (2017). Bioaccessibility, bioavailability, and anti-inflammatory effects of anthocyanins from purple root vegetables using mono- and co-culture cell models. Mol Nutr Food Res.

[CR36] Zhou C, Liu Z, Jiang J, Yu Y, Zhang Q (2012). Differential gene expression profiling of porcine epithelial cells infected with three enterotoxigenic Escherichia coli strains. BMC Genomics.

